# Blue justice: A survey for eliciting perceptions of environmental justice among coastal planners’ and small-scale fishers in Northern-Norway

**DOI:** 10.1371/journal.pone.0251467

**Published:** 2021-05-13

**Authors:** Sigrid Engen, Vera Helene Hausner, Georgina G. Gurney, Else Grete Broderstad, Rose Keller, Aase Kristine Lundberg, Francisco Javier Ancin Murguzur, Emma Salminen, Christopher M. Raymond, Jannike Falk-Andersson, Per Fauchald

**Affiliations:** 1 Norwegian Institute for Nature Research, Tromsø, Norway; 2 UIT–The Arctic Sustainability Lab, The Arctic University of Norway, Tromsø, Norway; 3 ARC Centre of Excellence for Coral Reef Studies, James Cook University, Townsville, Queensland, Australia; 4 Norwegian Institute for Nature Research, Lillehammer, Norway; 5 Nordland Research Institute, Bodø, Norway; 6 Helsinki Institute of Sustainability Science, University of Helsinki, Helsinki, Finland; 7 Department of Economics and Management, Faculty of Agriculture and Forestry, University of Helsinki, Helsinki, Finland; 8 Ecosystems and Environment Research Program, Faculty of Biological and Environmental Sciences, University of Helsinki, Helsinki, Finland; 9 Salt Lofoten AS, Tromsø, Norway; Swedish University of Agricultural Sciences and Swedish Institute for the Marine Environment, University of Gothenburg, SWEDEN

## Abstract

Ocean-based economic development arising from an increasing interest in the ‘blue economy’ is placing ecosystems and small-scale fisheries under pressure. The dominant policy response for dealing with multiple uses is the allocation of coastal space through coastal zone planning (CZP). Recent studies have shown that the rush to develop the blue economy and regulate coastal activity can result in social injustices and the exclusion of less powerful and unrecognized groups (e.g., small-scale fishers, women, Indigenous peoples and youth). To achieve a primary goal of the 2030 sustainable development agenda to “leave no one behind”, it is important to understand the implications of coastal planning and development for these groups. Here, we present a social survey protocol for examining perceptions of justice related to small-scale fisheries (SSF) in the context of the blue economy in coastal areas. Specifically, we designed the survey instrument and sampling protocol to assess whether decisions about the use of the coastal zone over the last five years have i) followed principles of good governance, ii) recognized fishers’ knowledge, culture and rights and iii) been attentive to impacts of changed coastal zone use on fisheries. The survey will engage coastal planners (N = app. 120) and fishers (N = app. 4300) in all the coastal municipalities (N = 81) in Northern-Norway. The sampling protocol is designed to ensure representation of different sectors of society, including those defined by gender, age, ethnicity and occupation (e.g., small-scale fishers, large-scale fishers, coastal planners).

## 1. Introduction

Increasing use and regulation of coastal areas necessitates heightened attention to justice and inclusivity in coastal governance, including the development of the blue economy [1–7]. Studies have shown how blue economy policies and the accompanying changes in rules and authority can lead to injustices, for example the spatial displacement of small-scale fishers and Indigenous peoples, exclusion from decision-making, and inequitable distribution of benefits and costs [7–12]. Here, we present a study protocol that explores the concept of blue justice in relation to the growth in the blue economy and coastal zone planning, specifically through a survey instrument designed to elicit key stakeholders’ perceptions of justice. The social survey protocol provides a theoretical and contextual background for our survey to ensure the validity, transparency and reproducibility of our results prior to data collection.

Attention to justice and inclusion for small-scale fisheries finds traction in a number of international policies and initiatives. At the first global conference on how to realize a sustainable blue economy in 2018, concerns related to small-scale fisheries, Indigenous peoples, women and youth were central [13]. Securing sustainable small-scale fisheries is specifically mentioned as a part of UN Sustainable Development Goal 14 (target b), while justice and inclusivity are covered by SDGs 10 and 16 (i.e., reduced inequalities and promoting peaceful and inclusive societies, respectively) [14]. The Ocean Panel (i.e., the high level panel for a sustainable ocean economy made up of 14 world leaders) identifies justice as one of five areas of transformation to secure a sustainable ocean economy, especially considering small-scale fishers, women, youth, coastal communities and Indigenous peoples [15]. The Food and Agriculture Organization’s (FAO) voluntary guidelines for small-scale fisheries are central in the work to “support the visibility, recognition and enhancement of the already important role of small-scale fisheries […]” [16] and the UN has declared 2022 as the international year of artisanal fisheries and aquaculture [17]. A justice framing has also been adopted by the To Big To Ignore (TBTI) Network, which is a global network of scientists focused on small-scale fisheries research [18].

The blue economy refers to the sustainable use of marine environments for economic activities, while blue growth can be thought of as the expansion of such activities [19–21]. However, definitions vary and the terms blue growth, blue economy and ocean economy are often used interchangeably [4, 8, 22]. Since the initial framing of the blue growth at the Rio +20 UN sustainability conference [22, 23], different narratives have been linked to the concept each with its own set of problems, solutions, actors and governance arrangements [24, 25]. In particular, critiques have been raised that blue growth (or blue economy) has become less about sustainability and more about capitalization of marine resources [8].

The term blue justice has been coined as a response to the restructuring of rules and authority over access, use and management of marine resources and marine space [9, 18]. Blue justice can be defined as a just and inclusive blue economy where recognitional, procedural and distributional justice concerns are at the forefront of the blue economy agenda [9]. A related term, blue degrowth, critiques capitalistic, growth driven policies and proposes participatory societal visions that emphasize coastal community rights and small-scale, local production and consumption as an alternative [26, 27].

Integrated coastal zone planning (CZP) is the regulation of the spatial and temporal distribution of human activities in coastal areas to achieve ecological, economic, and social objectives (i.e., a blue economy) [7, 28, 29]. It seeks the integration of multiple uses in the same planning framework to improve conflict management, facilitate the co-location of compatible activities and act as an alternative to sector-based approaches [29, 30]. CZP differs from the related concept marine spatial planning (MSP) because it focuses on the coastal zone and the integration of land-based and marine activities [29]. Ideally, CZP should be a form of adaptive management that is based on the best available knowledge (including local and indigenous knowledge) and where the early and continuous involvement of stakeholders is ensured [28, 29]. This has proved difficult to achieve in many cases, for instance with regards to the involvement of traditional knowledge holders like some small-scale fishers [10–12, 31].

There is no single globally agreed on definition of what is regarded as small-scale fisheries [32–35]. Both the academic literature and national policies rely heavily on technological indicators such as type of gear used or vessel length [32, 34]. The EU for instance refers to small-scale coastal fishing as fishing carried out by fishing vessels of an overall length of less than 12 meters [36]. Reducing a definition to vessel length precludes the variation and complexity in small-scale fishing [32]. The FAO loosely defines small-scale fisheries as the wide-ranging activities undertaken throughout the value chain by both men and women [16, 32], while Gibson and Sumaila [33] use vessel features (e.g., small vessels, passive gear, multi-gear, multi species etc.), economic features (e.g., low fuel consumption, little capital input, individual or community ownership) and social features (e.g., support social and cultural values, regulated partly through customary rules).

In the absence of a coherent understanding of blue justice, there is a critical need to elucidate the concept with respect to small-scale fishers, a key stakeholder group that is at risk of being marginalized by blue economy processes. Here, we present a quantitative survey approach [37–40] to examine stakeholders’ perceptions of justice with respect to the blue economy and coastal zone planning. Our approach is underpinned by a multidimensional [7, 41–45] and pluralistic view of justice (e.g., what is thought of as just by one actor can be unjust for someone else) [42, 45, 46].

We will survey small-scale fishers and coastal zone planners in coastal communities in Northern-Norway. In this region, small-scale fisheries have played an important role in coastal communities and been of immense cultural value for millennia [47]. Norway has a history of successful engagement and participation of fishers in fisheries management (e.g., quota setting, regulating fishery activity) [48, 49], and the viability of coastal communities has been an explicit objective of Norwegian fishery policy since the start [50]. However, the increased competition for coastal space spurred by growth in the blue economy, heightens the importance of attention to fishers’ involvement in CZP [51].

In the following sections of this paper we give a more detailed account of the different dimensions of justice in the context of CZP and small-scale fisheries (section 2.1), provide a detailed account of the Norwegian research context (section 3) and present the survey instrument (section 4) and the sampling protocol (section 5). We finish with a discussion of the strengths and weaknesses of this approach (section 6) and concluding remarks (section 7).

### 1.1. Blue justice for small-scale fisheries in coastal zone planning

A multidimensional view implies that justice is seen as “a balance of numerous interlinked elements of distribution, recognition and procedure” [43], and where none of the dimensions are reducible to another [7, 39]. A multidimensional view is the basis for other social justice surveys [37, 40] and the newly developed framework for the evaluation of the social justice of area-based marine management [7], supporting our choice of theoretical framework.

#### 1.1.1. Recognitional justice

Recognitional justice acknowledges the plurality of people’s values, identities, cultures, rights, institutions, knowledges and capabilities [38, 43, 52, 53]. It is a prerequisite for procedural and distributional justice [43, 46] and requires attention to the reasons behind poor distribution [41, 43, 54, 55]. Lack of recognition of small-scale fisheries in CZP can, for instance, entail viewing commercial fishers as one homogenous stakeholder group, which disregards the diversity of fishers’ interests, knowledges, culture, values and concerns. In particular, women are an important part of the workforce in fisheries (especially small-scale) [56–58], but often overlooked [58, 59]. Marine resources are also essential for the wellbeing of many Indigenous peoples, who often lack formal recognition of their marine tenure rights and the influence to protect their interests [60].

#### 1.1.2. Procedural justice

Procedural justice relates to the ways in which decisions are made: who is involved and has influence as well as where and when decisions happen [45]. As mentioned, CZP should integrate multiple uses, utilize the best available and pluralistic evidence, and ensure the early and continuous involvement of stakeholders in an interactive process [28, 29]. Nevertheless, many area-based marine management processes have superficial stakeholder involvement where decision structures are simply repackaged “… in the rhetoric of participation to legitimize the agendas of dominant actors” [10]. For example, Jay et al. [61] found that fishers recognized that pressures on fisheries only would increase as a result of the German Exclusive Economic Zone Plan, over which they had little influence. Nutters & Punto de Silva [12] reported insufficient involvement of fishers in MSP efforts in Rhode Island and Massachusetts in the USA. Fishers were frustrated about meeting location, poor timing, lack of capacity to participate and perceived lack of influence. Jones et al. [11] reviewed MSP cases in Europe and found they were based on top-down, ad hoc processes where sectoral objectives predominantly related to the national priorities of blue growth.

#### 1.1.3. Distributional justice

Distributional justice refers to the equity or fairness of distributions of benefits and burdens [45]. Although equality (i.e. equal distribution) is associated with justice, an equitable distribution can follow other principles, including needs or proportionality (according to, for example, effort or pre-existing rights) [3, 42]. Which of these principles constitutes distributional justice is likely to differ according to the situation (e.g. what is being distributed among whom) and who is deciding [62]. Justice can also imply not doing harm, mitigating and compensating for negative impacts or empowering already disadvantaged or vulnerable social groups [7, 39], e.g. using preferential treatment [16]. Distributional injustices arising from the blue economy and CZP include, for example, the spatial displacement of local users due to aquaculture and tourism expansion and negative impacts of pollution and waste disproportionally affecting certain groups [9].

## 2. The research context

The purpose of this section is to provide the reader with an account of the Norwegian research context to further understand the choice of survey questions, along with terms used in the survey and its framing.

### 2.1. Study area

Northern-Norway is currently viewed as a region of tremendous economic potential in blue industries [63, 64]. Favorable environmental conditions and technological innovation has made Norway a major player on the global marked for farmed fish [65] and further expansion in this industry is encouraged [64]. The region is also a tourism hub, attracting domestic and international visitors seeking to experience northern lights, marine wildlife and coastal communities [66, 67]. Energy, extractive industries and transportation are also on the rise [63]. Future prognoses point to large opportunities for growth and employment in Northern-Norway towards 2040 in both established (e.g., oil and gas, fishing, fish farming, and shipping) and establishing industries (e.g., coastal tourism, green energy, seabed mining and the utilizing new marine species) [68].

**Fig 1:**
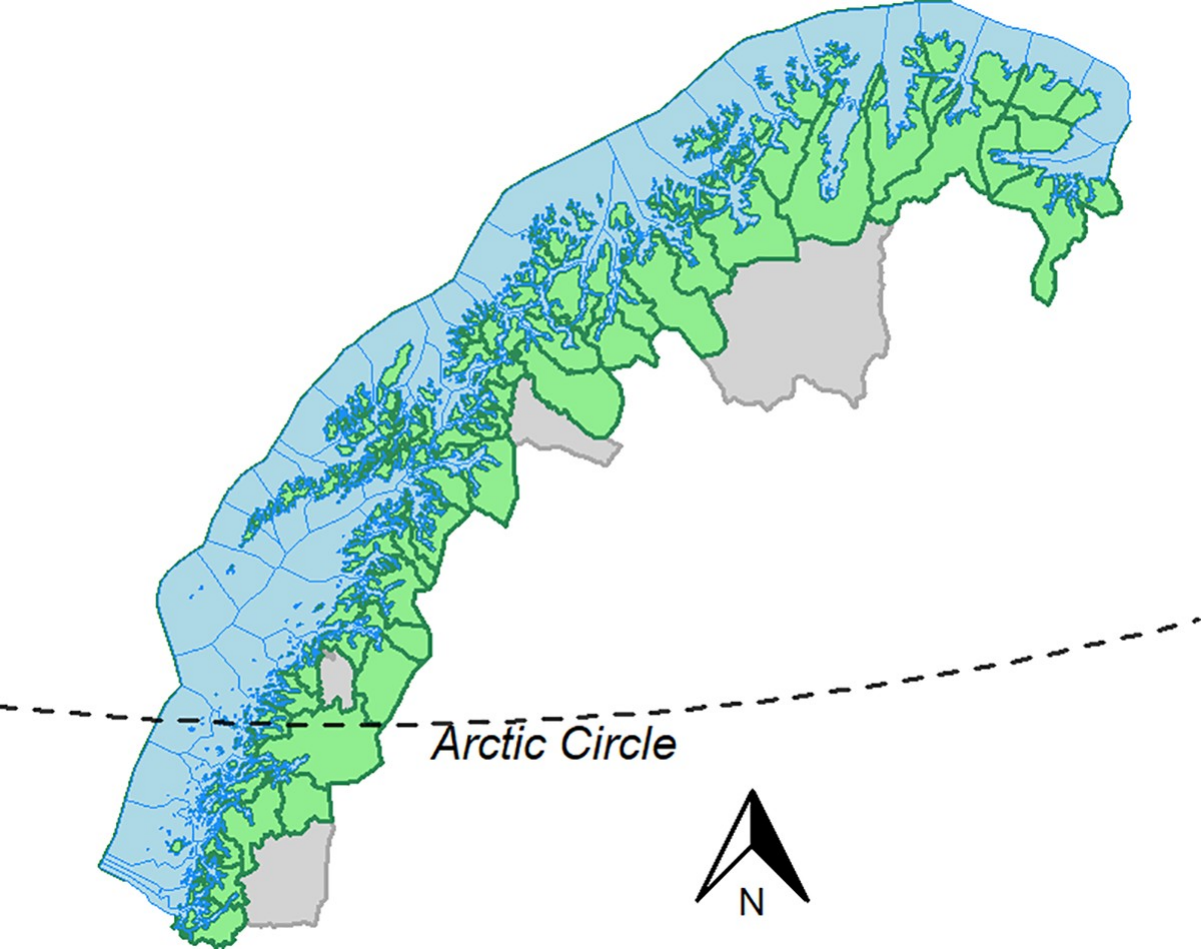
Study area. The 81 coastal communities of Northern-Norway (i.e., communities with a coastline). Ocean area in blue and land area in green.

Norway is also among the world’s top ten countries in terms of marine captures [57]. Approximately 2.5 million tons of fish, shrimp, scallops and shells were harvested in 2019 at a value of around 2 billion USD [69]. Northern-Norway is especially important for fisheries as almost half of all fishers in Norway live here [70]. The majority of Norway’s small coastal vessels are also found in this region [71], in addition to almost half of all the business within the seafood industry [64]. Our study area includes 81 coastal municipalities (i.e., municipalities with a coastline) in Northern-Norway (Fig 1). We excluded one municipality with a very small coastline (Beiarn) due to no registered fishers in 2020.

### 2.2. Coastal zone planning

Coastal planners have a key role in navigating tradeoffs between fisheries and other marine industries that are expanding as a result of growth in the blue economy. Coastal areas in Norway were traditionally used for fisheries and transportation and there were few conflicts between these two sectors [72]. It was the introduction of the aquaculture industry in the 1970s and especially the territorial conflicts between fishers and aquaculture that spurred the need for planning and zoning [72]. Since then, coastal zone planning has progressed differently in Norwegian municipalities. In 2018 some municipalities had updated plans of good quality, while others had barely started or had old and outdated plans [73].

Municipalities in Norway are in charge of making legally binding plans for their own land area and the coastal area delineated by 1 nautical mile from the baseline (Planning and Building Act §1–2). Coastal plans should coordinate activity, weigh different interests against each other, stipulate the future use of coastal space and minimize conflicts [74]. Cooperation at a regional level (through for instance intermunicipal coastal planning) should be considered, especially if municipal borders divide fjords or bays [73, 74]. Coastal plans can be made in conjunction with the land use plan or as a separate plan for the coastal zone (Planning and Building Act, 2008, § 11–1). In the latter case the proposed use of coastal space and resources should take into consideration the activities on land [74].

Local autonomy is restricted by sectoral legislation relating to for instance fisheries or aquaculture regulations (e.g., Marine Resource Act, 2008, Aquaculture Act, 2005) or national and regional concerns. State or regional authorities such as the Coastal Affairs Directorate, the Fisheries Directorate, The Food Safety Authority, the County Municipality and the County Governor can object to coastal plans in matters of national or regional concern or that for other reasons are of importance to the agency’s case administration (Planning and Building Act § 5–4). The Sámi are the indigenous people of northern and middle Fennoscandia [75] and the Sami Parliament (i.e., the democratically elected body of the Sámi people in Norway) can object in matters of importance for Sámi culture or commercial activity (Planning and Building Act § 5–4). Other municipalities can object in matters of importance for the municipality (Planning and Building Act § 5–4).

It is a legal requirement that coastal zone planning in Norway is open, transparent, inclusive, participatory and deliberative [76] and stakeholder involvement is a requirement (Planning and Building Act, 2008, § 5–1). The government has issued guidelines pertaining to stakeholder involvement in municipal planning that among other things stipulate the municipality should make efforts to involve groups that are affected by coastal zone planning and management, but who are less able or willing to participate (e.g. young people, older people, national minorities, Indigenous peoples, people with bad prior experience from participation) [77]. However, coastal planners can come from different units in the municipality (e.g. environmental protection unit or industrial development unit) which could influence who they consider relevant to involve or not [76]. A lack of capacity and competence in the municipality may also make CZP challenging, especially in small municipalities where local planners have multiple administrative responsibilities in addition to CZP [78]. The capacity of stakeholders to participate in small municipalities may also be limited, i.e. lacking a strong regional coordinating organization for local interests, such as Sámi [79]. Fishers in general are recognized as key stakeholders by planners in Norway, however less is known about the involvement of different groups of fishers such as small-scale, female or indigenous fishers [76, 80].

### 2.3. Fisheries management

Fishery management in Norway has developed from an open-access fisheries to a closed system regulated with partly transferable vessel quotas and permits [81]. While local governments in Norway have decision-making power over the use of the coastal zone, Norwegian fishery management is centralized. It is first and foremost the norms, values and principles that the national state represents that are decisive in how rights are distributed [82]. International monitoring and stock assessments carried out by ICES–The International Council for the Exploration of the Seas, along with annual fisheries negotiations between Norway and other parties, determine the national quotas (Total Allowable Catch—TAC) available for distribution when it comes to stocks that are shared with other countries [83].

Each year the Norwegian Ministry for Trade, Industry and Fisheries determines the distribution of the TAC to groups of vessels and individual vessels. These regulations are based on proposals drafted by the Norwegian Fisheries Directorate, after being discussed at annual meetings with fisheries organizations, the fishing industry, the Sami Parliament, local authorities, environmental organizations and other stakeholders [84]. The stakeholders at these meetings have a an advisor and not an actor status, which means they do not have direct influence on the final decisions [82].

Fisheries in Norway are divided into many groups for the purpose of regulating access to fisheries. First, a distinction is made between the benthic (cod, saithe, haddock, shrimp, crab, lobster) and the pelagic (e.g., mackerel, herring, blue whiting, capelin) fisheries [85]. Then the fleet is divided into coastal and offshore (i.e., vessels with an coastal or offshore license, respectively), followed by a further division into groups based on the species or populations harvested, the gear used and vessel size [85]. For instance, there are four regulatory groups for fishing cod north of 62°N, namely closed group coastal fisheries, open group coastal fisheries, trawl and conventional offshore vessels [86]. The closed coastal group is further divided into groups based on vessel length (less than 11m, 11–14.99m, 15–20.99m and 21–27.99m) [86].

The open group coastal fisheries is assigned quotas in many important closed, quota-regulated fisheries (e.g., cod, saithe and haddock north of 62°N, Norwegian spring spawning herring, mackerel and king crab) [87]. The purpose of the open group fisheries is to allow for part-time commercial fishers, recruit young people as vessel owners, secure the material basis for Sámi culture, and allow retired commercial fishers to downscale their activity [87]. It should in principle be possible for anyone to participate at low costs in the open group fisheries [87]. A condition for participation in the open group fisheries for cod, saithe and haddock north of 62°N is that the vessel must be smaller than 11 meters [50].

Fisheries in Norway are, in addition to extensive formal regulations, also organized through informal rules that are a part of coastal culture. These rules originate from competitive practices where fishers’ knowledge, social relations and status play a role in determining access to local fishing grounds [88, 89]. The rules are created and altered through ongoing debates among fishers and enforced through social control [88, 89]. Jentoft and Buanes [90] argue that community norms and enforcement mechanisms are essential institutions that should be actively supported and not only assumed in coastal management.

### 2.4. Diversity in Norwegian fisheries

**Fig 2:**
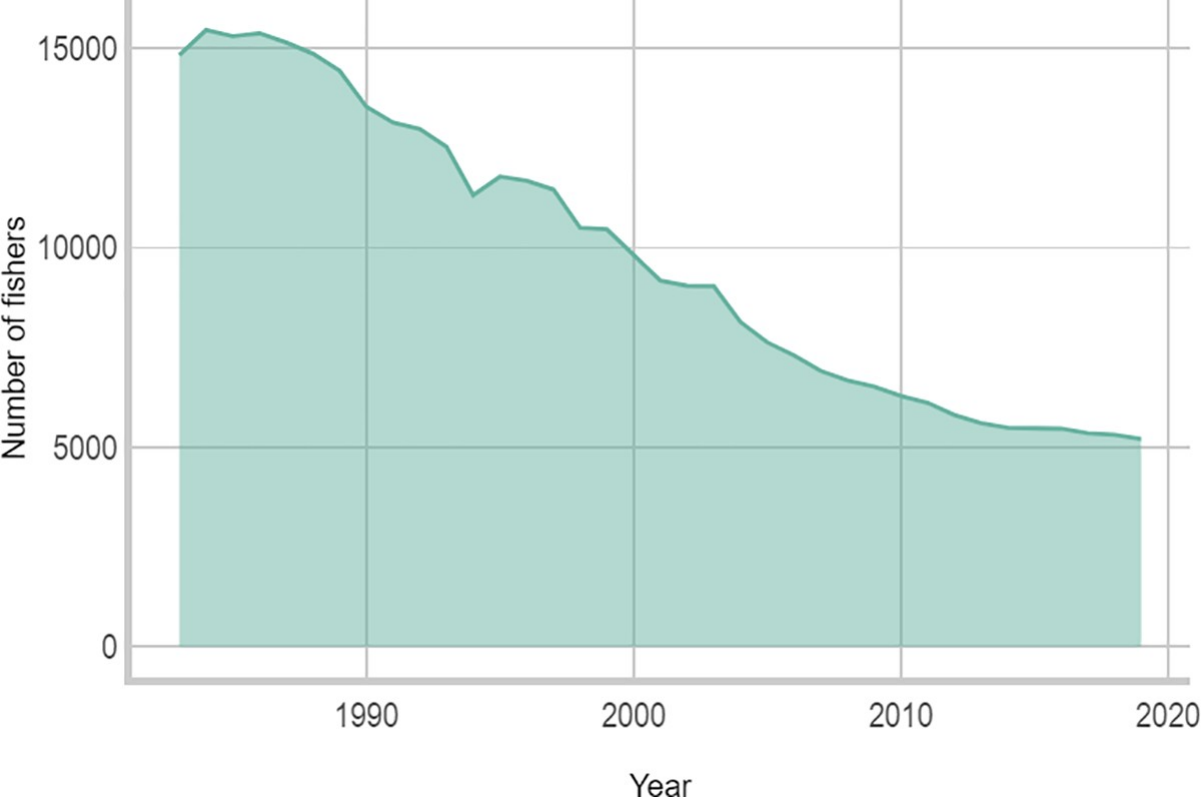
Number of fishers. The number of registered fishers in Northern-Norway (1983–2019) (Data source: The Norwegian Fisheries Directorate).

The number of fishers in Norway has declined steadily since 1950 and so has the number of vessels [91] (Fig 2). Technological efficiency has increased the amount of fish each fisher can catch. This increased efficiency along with a higher living standard and a subsequent need for providing competitive wages to ensure recruitment to fisheries, have been major drivers behind the reduction in fishers and vessels [92].

#### 2.4.1. Small-scale operators

While Norwegian fishery policy has aimed at increasing efficiency and profitability, it has at the same time tried to protect the small-scale fishers segment from market forces, by for instance, restricting the opportunities for consolidating quotas among vessels below 11 meters [93]. Today, vessels below 11 meters make up around 80% of the Norwegian fishing fleet [94]. These smaller, less mobile operators are particularly important for the local small-scale fishing industry [87], and thus also for the geographical spread of fishing activity in Norway.

However, despite the fact that small vessels make up a large portion of the fishing fleet in Norway, the trend is towards larger and more mobile vessels with weaker ties to coastal communities, an increased number of larger and a reduced number of smaller fish industry businesses and fish landing sites, a reduced number of municipalities with fish industry and concentration of quotas on fewer actors [87, 95].

#### 2.4.2. Female fishers

In Norway, female fishers have represented between 2.7 and 3.5% of all fishers since 1990 [58, 96]. In 2019 there were 329 registered female fishers with fishing as the main occupation [97]. Most professional fishers and owners of fishing vessels and quotas are men [96]. Female fishers earn less than the male fishers [98]. A significantly lower number of gender equality measures have been implemented in fisheries compared with other industries in the country [96]. Moreover, female fishers are rarely mentioned in public assessments and white papers [96].

#### 2.4.3. Indigenous fishers

The Sea Sámi refers to Sámi people who live along the coast and in the fjords, primarily in Northern-Norway. Some of them descend from the Sea Sámi population who had fishing and farming as livelihoods [99]. Small-scale fishing is an important part of the material basis for Sámi culture [81], and securing natural resources for Sámi livelihoods and culture is a goal of the main acts governing coastal planning and marine resources in Norway (i.e., Planning and Building Act (2008), Nature Diversity Act (2009), Marine Resource Act (2008) and the Participatory Act (1999)). Moreover, the Norwegian Constitution § 108 requires that Norwegian authorities enable the protection and continued development of Sámi culture, livelihood activities and community life. Nevertheless, Sea Sámi areas struggle with population decline, partly as a result of problems within fisheries [100]. According to the Norwegian Standing Committee on Scrutiny and Constitutional Affairs it is a governmental responsibility to ensure a fair distribution of quota along the coast to secure viable coastal communities.

Many inquiries have been made about strengthening coastal and fjord fishing in Sea Sámi areas [100]. In a recent investigation, the Coastal Fisheries Committee was tasked with exploring whether the Sámi had similar historical rights to marine space and marine resources as they have on land [60]. The conclusion was that fishers living in Finnmark have a historical right to fish and that this right should be legally recognized and formally implemented through a regional co-management system [60, 82, 100]. National authorities did not support this conclusion as they regard the current regulations for participation in fisheries to be in accordance with the international obligations for the Sámi as a minority and an indigenous people [82]. As an alternative the Fjord Fisheries Board for Northern-Norway was established [60]. The purpose of the board is to strengthen the management of the fjord fisheries, especially taking into consideration Sámi use and Sámi communities. The Fjord Fisheries Board is, however, limited in their mandate, which does not allow for autonomous decision-making powers for Indigenous peoples–in conflict with international law [60].

The Sami Parliament plays an important role in protecting the rights and voicing the concerns of Sámi small-scale fishers [101]. They distribute investment funds within Sámi areas (so-called Sámi Fishing Rights (SFR) areas) enabling residents in these areas to apply for financial support for small-scale fisheries (e.g., for fish landing sites, fish processing, fishing vessels, fishing gear and equipment, recruitment measures) [102]. These funds have enabled growth in the fishing activity in some Sea Sámi areas [101]. The Sami Parliament should be consulted by national authorities in matters of importance to the Sámi. They have issued planning guidelines that stipulate that coastal plans should protect traditional fishing and spawning grounds of importance for fisheries in Sámi coastal areas and fjords and ensure that changes to the use of space do not result in irreversible damage to fisheries of local importance [103]. The guidelines also list potential stakeholders.

Several studies have reported the lack of representation of Sámi perspectives in CZP and a lack of knowledge regarding the impact of changes in the use of the coastal zone on Sámi culture [76, 78].

### 2.5. Fishers’ knowledge

Fishers’ knowledge can be defined as knowledge and perceptions about the fishery sector, including local, ecological, technological or management related issues [104]. This knowledge is important for fishers’ ability to anticipate and deal with changes [105–107]. Indeed, fishers are continuously adapting to environmental and socioeconomic changes. Fishers’ knowledge is also of value for sustainable fishing practices e.g., knowing how to avoid vulnerable habitat or the entanglement and subsequent loss of fishing gear.

Historically, fishers’ knowledge has been important in fisheries management in Norway, especially pertaining to zoning efforts to avoid conflict between different types of fishing gear [104]. The use of fishers’ knowledge in modern fisheries management has been met with more reluctance [108]. Today, fishers’ knowledge in Norway is incorporated into national fishery management through the Institute of Marine Research’s (IMR) Reference Fleet. This is a small group of fishing vessels paid to provide the IMR with detailed information about their fishing activity and catches on a regular basis [108]. Fishers are not involved in the interpretation of the data or in the subsequent quota setting [104].

Fishers’ knowledge is also documented and made available for planners, managers and the general public in the form of online maps showing the location of fishing areas, nursing areas, spawning areas, important areas for shrimp, netpen sites etc. The maps are produced by the Fisheries Directorate and are based on interviews with fishers. The information is validated through scientific assessments conducted by the IMR [104]. However, these maps have in some cases been found to be inadequate, not updated or quality assured [109]. Furthermore, fishing areas, spawning areas and nursing areas often have a diffuse delineation and can be difficult to map, and some fishers may not be willing to disclose where they fish [104].

Fishers’ knowledge may also be elicited through direct involvement of fishers in coastal planning and management [76]. For instance, Hersoug et al. [78] found that local involvement in an intermunicipal planning process in Northern Norway contributed with substantial local knowledge about ecosystem services in the coastal zone. Local involvement and mapping of important places also reduced the level of conflict.

## 3. The blue justice survey

We developed the survey based on existing justice theory and frameworks [7, 37, 39, 40, 110, 111], a review of the literature related to coastal zone planning and fisheries in Norway (see section 3), along with input from researchers in the project with experience from fisheries management, coastal planning and justice theory. We investigated the face validity of the survey by piloting it with people who have worked with planning and management in Norway, along with employees at the Fisheries Directorate, fishers’ organizations, fishers and other researchers with experience from fisheries related research in Norway.

In our survey we capture the recognitional, procedural and distributional elements of blue justice using 17 indicators (Table 1 and S1 and S2 Tables). Participants will be asked to rate 17 blue justice statements on a five-point Likert scale (very small or no degree to very large degree). A no-opinion/ not relevant option is also included. The blue justice questions focus on the last five years to make our enquiry time and context specific. This also increases the relevance of the survey for management. We chose a five-year time frame as it is a long enough period for changes to have occurred, but at the same time a short enough time period for people to remember or having experienced changes that have taken place (e.g., planners who recently moved to the area or young fishers). With regard to distributional justice we only focus on impacts on fisheries to avoid making the survey too long, while Bennett et al. [37] also asked about impacts on the community and points to the need for assessing the presence of mitigation and compensation mechanisms [110].

**Table 1:** Survey indicators of blue justice for small-scale fisheries in coastal zone planning and management.

**Recognitional justice**	
*Attribute*	*Indicator*
	*Focus on your municipality the last 5 years*. *Regarding decisions about the use of the coastal zone*, *to what degree has/have*
Knowledge^1^^,^^2^	Fishers’ knowledge been utilized?
Culture^1^^,^^2^	The importance of the coastal fishing culture been recognized?
Rights^1^^,^^2^	Matters of importance to the Sea Sámi been considered?
**Procedural justice**	
*Attribute*	*Indicator*
	*Focus on your municipality the last 5 years*. *To what degree has/have*
Participation^1^	Fishers had the opportunity to participate in decisions about the use of the coastal zone?
Influence^1^^,^^2^	Fishers had influence over decisions about the use of the coastal zone?
Access to justice^1^^,^^2^	Conflicts between fishers and other users of the coastal zone been resolved?
Accountability^1^^,^^2^	Fishers known who to contact when use of the coastal zone has caused challenges for fisheries?
Trust^1^^,^^3^	There been trust between fishers and those in charge of coastal zone planning?
Fairness^2^^,^^3^	Decision-making about the use of the coastal zone been fair?
**Distributional justice**	
*Attribute*	*Indicator*
	*Focus on your municipality the last 5 years*. *To what degree has/have changes in the use of the coastal zone*
Marine resource abundance^1^^,^^3^	Reduced the number of fish and shellfish?
Important habitat	Negatively influenced important habitat for fisheries?
Physical access	Restricted fishers’ access to fishing grounds?
Livelihood^1^	Reduced the number of fishers?
Fishers’ income^1^	Reduced fishers’ income?
Quality of fish or shellfish	Reduced the quality of fish or shellfish?
Fishing effort^3^	Increased time, effort and/or travel distance during fishing?
Fairness^3^	*Focus on your municipality the last 5 years*. *To what degree has/have* The distribution of positive and negative impacts from coastal zone management been fair?

^1^Bennett et al. [37]

^2^Zafra-Calvo et al. [40]

^3^ Gurney et al. [111].

In addition, participants will be asked to identify the three most important challenges for fisheries in their municipality today and in 2050. They will also be asked to identify which changes to the use of the coastal zone have had a negative impact on fisheries the last five years and which have had a positive impact.

Finally, we included questions related to the background of coastal planners (e.g., ties to fisheries, position in the municipality) and fishers (e.g., questions to related to fishing gear use, resource dependency and income; Table 2). We also ask planners about the coastal zone plan status in their municipality.

**Table 2:** Participant demographics and other characteristics potentially related to perceptions of blue justice.

Planners:	Fishers:
Municipality	Municipality
Gender	Gender
Age	Age
Formal education	Formal education
Position (in municipal administration)	Position (on the fishing vessel)
Work experience	Work experience
Years in municipality	
Personal ties to fisheries	
Coastal plan status	
Intermunicipal coastal plan status	
	Ethnicity
	Income
	Resource dependency
	Characteristics of fishery (e.g., size of fishing vessel, species harvested, type of gear used)
	Family involvement in fisheries

In the survey we refer to growth in the blue economy as *changes in the use of the coastal zone* and coastal zone planning as *decisions about the use of the coastal zone*. We will identify which changes in the use of the coastal zone impacts fisheries and how this is related to growth in the blue economy in the separate questions already mentioned. We can also look at associations between perceptions of justice and the blue economy using other types of data (e. g., aquaculture production in the municipality, number of visitors etc).

We use the term “fisheries” throughout the survey as opposed to small-scale fisheries, local fisheries, or coastal fisheries for inclusivity. We will survey all the fishers in the region and will make a distinction between perceptions of justice for the different fisheries by looking at fishers’ backgrounds.

We will ask planners to base their answers on the municipality that they work in, while fishers will be asked to choose the municipality that they have the most knowledge about and/or attachment to. Many fishers in Norway travel the coast to take advantage of the opportunities for rich fisheries in different parts of the country at different times of year. Thus, the municipality that they are registered in is not necessarily the municipality that is most important for their fishery.

## 4. Sampling protocol

### 4.1. Study participants

The study participants include coastal planners and fishers in the 81 coastal municipalities in Northern Norway (see study area). We obtained contact information for coastal planners (n = approx.120) by contacting the County Governor and the County Municipality. We also searched the municipalities’ web-pages to check and update the lists provided.

The Fisheries Directorate publishes a list over all registered fishers online. We downloaded this list of names of all fishers and the municipality that they are registered for Northern-Norway in October 2020. The list contained a total of 5151 fishers.

It is possible to be an active fisher without being registered, for instance as a minority owner of- or as crew on a fishing vessel. However, most of the majority owners of fishing vessels are registered [87]. All the fishers in the Fisheries Directorate’s database were registered in one of these 81 coastal municipalities except one fisher registered in the Hattfjelldal municipality.

**Fig 3:**
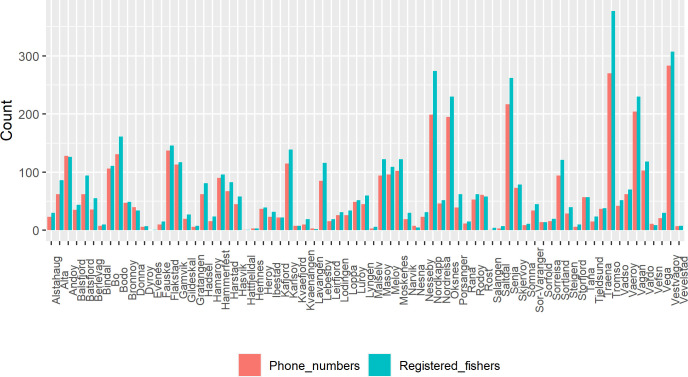
Fisher’s phones: Counts of registered fishers (n = 5151) and fishers’ phone numbers (n = 4278) by municipality.

We subsequently used this list to search the internet for phone numbers in a semi-automated manner: we used R [112] to search a Norwegian telephone database (www.gulesider.no) and automatically query every name on the list. If only one person matched the expected postal code, all the available information was downloaded (i.e. telephone numbers and full address). No data was downloaded if several matches were retrieved for a given name in the same municipality or if the name had an associated company. This reduced the likelihood of erroneously sending the survey to the wrong participants. We were able to retrieve contact information for 4278 fishers. The distribution of retrieved phone numbers and the number of fishers by municipality seem to correspond well (Fig 3).

### 4.2. Recruitment

We will send our web-based survey to coastal planners and fishers in Northern-Norway in April/May 2021. We will encourage the planners to forward the survey to other people in the municipal administration that they consider relevant participants. Coastal planners will receive an email with an invitation to participate and a link to access the survey, whereas fishers will receive a text message with a similar invitation and a survey link. We chose to use text messages to contact fishers to make it easy for them to respond by simply clinking on the link to access the survey using their smart phone as we have no means of obtaining the fishers´ email addresses. Alternatively, we could have obtained their postal addresses and sent the survey invitation as a letter. However, this might make some more reluctant to respond because they have to manually type the link they receive into the browser in order to access the survey. Participants will have three weeks to complete the survey. We will send two reminders to those who have not replied–the first after one week and the second after two weeks.

We will use the software SurveyXact https://www.surveyxact.com/ which is produced by Rambøll Management Consulting. This is a highly used tool by governmental and private organizations in Scandinavia.

### 4.3. Human subjects research ethics and data protection

An ethical review of our project (# 364014) has been conducted by the Norwegian Centre for Research Data (NSD) which is the Data Protection Official for Research for all the Norwegian universities and research institutes. The NSD has evaluate has evaluated whether our survey is compliant with the EU General Data Protection Regulation 2016/679 (GDPR). Participants have to sign a consent form to participate in the study. This form states the purpose of the study, informs that participation is completely voluntary and that participants can withdraw from the study at any time. It also explains how the data is stored and reported, and that participation is anonymous. We also provide contact details and encourage participants to notify us or NSD about any concerns.

## 5. Strengths and weaknesses of a social survey approach

**Fig 4:**
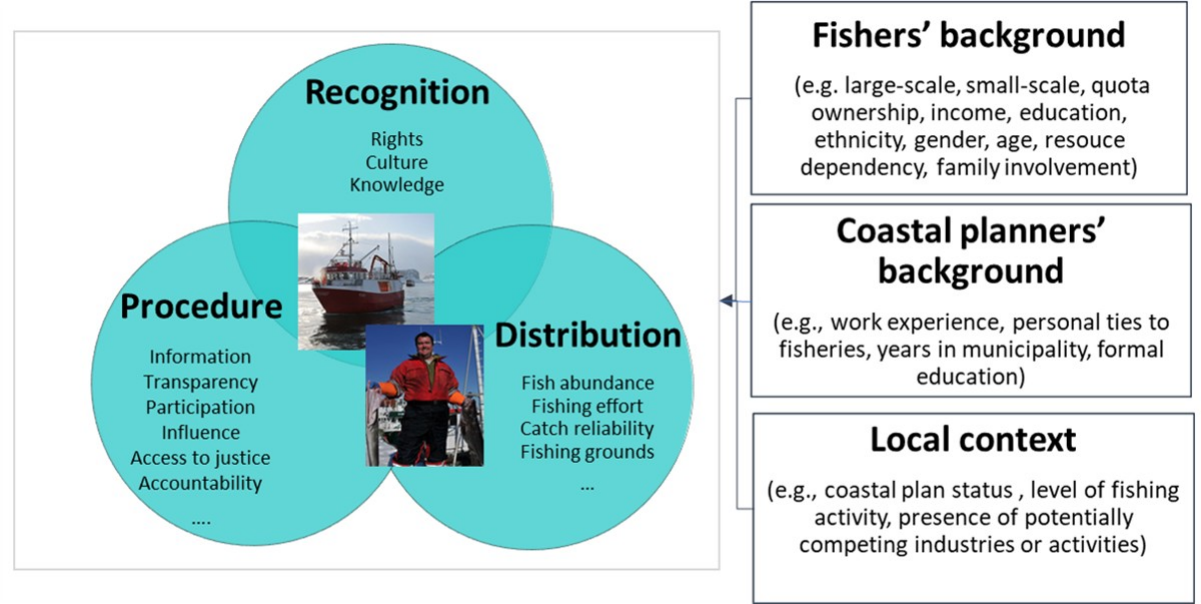
Illustrating associations that can be explored statistically using a social survey approach (e.g., the associations between perceptions of justice, coastal planners’ and fishers’ background and local contextual variables). Photos by the Norwegian Society for Sea Rescue, Flickr under a CC BY-NC-SA 2.0 license.

As with any method, our approach has strengths and weaknesses. A key strength of our approach is the anticipated large sample size collected over a large spatial area. Such empirical data enables comparative analyses, statistical representation and potentially the generalization of results. For example, we can examine whether relationships vary for different sectors of society (e.g. male and female fishers), including with an intersectional lens (e.g. male and female fishers in different age groups). Furthermore, quantitative survey data can be combined with other types of quantitative data (e.g., the number of fishers in the community, population size, the presence of fishing industry, the size of fish landings) for assessing the influence of such factors in explaining perceptions of justice and potentially discover associations that may not be self-evident (Fig 4).

There are several weaknesses and potential challenges of our approach. First, an important weakness of large-*N* surveys is that it entails a reductionist approach by necessitating a small number of questions with largely closed-ended responses. This can privilege issues or characteristics that are more easily quantified than those that are not (e.g. power) and potentially lead to the over- simplification and omission of issues that are important in a particular place [111]. Indeed, our survey based on pre-determined components of justice curtails respondents’ conceptualizations of justice. In contrast, qualitative inductive approaches may better capture how justice is conceptualized in a particular place (e.g. Lau et al. [113]). This trade-offs between generalizability and case-based relevancy (including use of qualitative interview approaches) is well-recognized in the literature (e.g. Cox et al. [114], Gurney et al. [111]).

Further challenges of our approach includes the risk of low response rates and responses that are skewed towards a certain segment of the population studied (e.g., those well-educated, most knowledgeable, who have a high income). Additionally, given we plan to only survey fishers and planners, we are overlooking the perspectives of other important stakeholders (e. g., fish farming, tourism, recreation), the views of which are critical to understand to gain a complete picture of blue justice.

## 6. Conclusion

We have developed a survey instrument that will allow the collection self-reported measures of blue justice among fishers and planners. The empirical data collected using this survey instrument can inform coastal zone planning and management for the just allocation of benefits and burdens, improved recognition of diverse groups within fisheries as well as new procedures for engaging stakeholders.

## Supporting information

S1 Table Survey to coastal planners. (DOCX)

S2 Table Survey to fishers. (DOCX)
