# Supplementary material for: Blue justice: A survey for eliciting perceptions of environmental justice among coastal planners’ and small-scale fishers in Northern-Norway
Source: PLoS One. 2021 May 13;16(5):e0251467. doi: 10.1371/journal.pone.0251467 (PMC8118289; doi:10.1371/journal.pone.0251467)
Supplement: S1 Table — (DOCX) [file pone.0251467.s001.docx]

S1 Table. Blue justice survey to coastal planners. Questions are arranged in the order they appear for the participant.

|  |  |  |  |  |
| --- | --- | --- | --- | --- |
| *Topic* | *Survey Questions (English)* | *Potential Responses (English)* | *Survey Questions (Norwegian)* | *Potential Responses (Norw.)* |
| **Municipality** | Which municipality do you belong to? | (drop down list of the 81 coastal municipalities in Northern-Norway) | Hvilken kommune tilhører du? | (nedtrekksliste over de 81 kystkommunene i Nord-Norge) |
| **Recognitional justice:**  Knowledge  Culture  Rights | *Focus on your municipality the last 5 years:*  *Regarding decisions about the use of the coastal zone – to what degree has/have:*   - Fishers’ knowledge been utilized - The significance of the coastal fishing culture been recognized - Matters of importance to the Sea Sami been considered - You used the Sami Parliament planning guidelines   *To what degree have/has:*   - Younger fishers (18-25 years) had the opportunity to choose fishing as an occupation   *(the latter question is placed among the procedural justice questions in the survey)* | Very small (or no) degree (= --) small degree (= -) / some degree (=N)/ large degree (= +) / very large degree (= ++) / not relevant or don’t know (1-6) | *Fokuser på din kommune de siste 5 årene:*  *Angående beslutninger om bruk av kystsonen - i hvor stor eller liten grad har:*   - Fiskeres kunnskap blitt benyttet - Betydningen av kystfiskekulturen blitt anerkjent - Det blitt tatt hensyn til sjøsamiske forhold - Du du benyttet Sametingets planveileder   *I hvor stor eller liten grad har:*   - Yngre fiskere (18-25 år) hatt muligheten til å velge fiske som karrierevei   *(det siste spørsmålet er plassert blant prosess spørsmålene i undersøkelsen)* | Svært liten (eller ingen) grad (= --) liten grad (= -) / noen grad (=N)/ stor grad (= +) / veldig stor grad (= ++) / ikke aktuelt eller vet ikke (1-6) |
| **Distributional justice:**  Fish abundance  Access to fish grounds  Habitat  Livelihood  Quality of catch  Fishing effort  Income  Fairness | *Focus on your municipality the last 5 years:*  *To what degree have changes in the use of the coastal zone:*   - Reduced fish and shellfish abundance - Restricted fishers’ access to fishing grounds - Negatively influenced important habitat for fisheries - Reduced the number of fishers - Reduced the quality of fish or shellfish - Increased time, effort and/or travel distance during fishing - Reduced fishers’ income   *To what degree has:*   - The distribution of positive and negative impacts from coastal zone management been fair   *(the latter question is placed among the procedural justice questions in the survey)* | Very small (or no) degree (= --) small degree (= -) / some degree (=N)/ large degree (= +) / very large degree (= ++) / not relevant or don’t know (1-6) | *Fokuser på din kommune de siste 5 årene:*  *I hvor stor eller liten grad har endringer i bruk av kystsonen:*   - Redusert mengde fisk og skalldyr - Begrenset fiskeres tilgang til fiskeområder - Hatt en negativ påvirkningen på viktige habitat for fiskeri - Ført til færre fiskere - Redusert kvaliteten på fisk eller skalldyr - Ført til økt tidsbruk, innsats og/eller reiseavstand under fiske - Redusert fiskeres inntekt   *I hvor stor eller liten grad har:*   - Fordelingen av positive og negative effekter av kystsoneforvaltning vært rettferdig   *(det siste spørsmålet er plassert blant prosess spørsmålene i undersøkelsen)* | Svært liten (eller ingen) grad (= --) liten grad (= -) / noen grad (=N)/ stor grad (= +) / veldig stor grad (= ++) / ikke aktuelt eller vet ikke (1-6) |
| **Procedural justice:**  Transparency  Accountability  Influence  Access to justice  Trust  Fairness | *Focus on your municipality the last 5 years:*  *To what degree have/has:*   - Fishers had the opportunity to participate in decisions about the use of the coastal zone - Fishers known who to contact when the use of the coastal zone has caused problems for fisheries - Fishers had influence in decisions about the use of the coastal zone - Conflicts between fishers and other users of the coastal zone been resolved - There been trust between fishers and those in charge of coastal zone planning - The way decisions about the coastal zone has been made been fair | Very small (or no) degree (= --) small degree (= -) / some degree (=N)/ large degree (= +) / very large degree (= ++) / not relevant or don’t know (1-6) | *Fokuser på din kommune de siste 5 årene:*  *I hvor stor eller liten grad har:*   - Det blitt lagt til rette for fiskernes medvirkning i beslutninger om bruk av kystsonen - Fiskere visst hvem de skal kontakte når bruk av kystsonen har ført til problemer for fiskeri - Fiskere hatt innflytelse i beslutninger om bruk av kystsonen - Konflikter mellom fiskeri og andre brukere av kystsonen blitt løst - Det vært tillitt mellom fiskere og de med ansvar for kystsoneplanlegging - Måten beslutninger om bruk av kystsonen har blitt tatt på vært rettferdig | Svært liten (eller ingen) grad (= --) liten grad (= -) / noen grad (=N)/ stor grad (= +) / veldig stor grad (= ++) / ikke aktuelt eller vet ikke (1-6) |
| **Challenges for fisheries today** | What are the three greatest challenges for fisheries in your municipality today? | (participants are asked to list three challenges) | Hva er de tre største utfordringene for fiskeriaktiviteten i din kommune i dag? | (deltakerne bes om å liste tre utfordringer) |
| **Challenges for fisheries in 2050** | In 30 years - what are the three greatest challenges for fisheries in your municipality then? | (participants are asked to list three challenges) | Om 30 år - hva er de tre største utfordringene for fiskeriaktivitet i din kommune da? | (deltakerne bes om å liste tre utfordringer) |
| **Changes in the use of the coastal zone** | *Focus on your municipality the last 5 years:*   - What changes in the use of the coastal zone have had a negative impact on the fisheries? - What changes in the use of the coastal zone have had a positive impact on the fisheries? | (open question) | *Fokuser på din kommune de siste 5 årene:*   - Hvilke endringer i bruk av kystsonen har hatt negativ innvirkning på fiskeriaktiviteten? - Hvilke endringer i bruk av kystsonen har hatt positiv innvirkning på fiskeriaktiviteten? | (åpent spørsmål) |
|  | Do you have experience with Sea Sami matters in coastal zone management?  *(follow up question placed below the recognitional justice questions in the survey)* | Yes/No | Har du erfaring med sjøsamiske forhold i kystsoneforvaltninga?  *(oppfølgingsspørsmål plassert etter spørsmålene om anerkjennelse)* | Ja/nei |
|  | *If the respondent answers yes to the former question the following question is also asked:*  What are your experiences with the consideration of Sea Sami matters in coastal zone management? | (open question) | *Hvis respondenten svarer ja på forrige spørsmål får vedkomne også spørsmål om:*  Hvilke erfaringer har du med om det tas hensyn til sjøsamiske forhold i kystsoneforvaltninga? | (åpent spørsmål) |
| **Education** | What is your highest level of education? | No education or not finished primary school / Primary & or secondary school / High School / Vocational training / Higher education: University and College education, lower level (Bachelor's degree) / Higher education: University and College education, higher level (Master's degree, PhD degree, Post graduate university degree) | Hva er din høyeste utdanning? | Ingen utdannelse eller ikke fullført grunnskole / Grunnskole (barneskole, undomsskole, folkeskole, realskole, framhaldsskole) / Videregående skole, gymnas / Fagskole (yrkesrettet alternativ til høyskole eller universitet) / Universitets- og høgskoleutdanning, lavere nivå (inkl. bachelor, cand.mag.) / Universitets- og høgskoleutdanning, høyere nivå (inkl. master, hovedfag og forskerutdanning) |
| **Gender** | What is your gender? | Male/female/other or do not wish to respond | Hva er ditt kjønn? | Mann / kvinne / annet eller ønsker ikke å svare |
| **Age** | What is your age? | 18-24/ 25-29/ 30-39/ 40-49 / 50-59 / 60-66 / 67-69 / 70+ | Hva er din alder? | (same as English version) |
| **Years in municipality** | How many years have you lived in the municipality where you work or in the surrounding area? | List # of years | Hvor mange år har du bodd i kommunen du jobber i, eller i omkringliggende områder? | (same as English version) |
| **Ties to fisheries** | Do you have any personal ties to fisheries (for instance have worked in the industry) ? | Yes /No | Har du en personlig tilknytning til fiskerinæringen (f.eks. har arbeidet i næringa)? | Ja / Nei |
| **Position** | What is your position in the municipality? | (open question) | Hva er din stilling i kommunen? | (same as English version) |
| **Experience** | How many years have you worked with planning and management? | List # of years | Hvor mange år har du jobbet med planlegging og forvaltning? | (same as English version) |
| **Status coastal plan** | Does your municipality have a coastal zone plan? | The plan is under development / Yes, the plan is between 0-5 years old / Yes, the plan is between 6-10 years old / Yes, the plan is between 11-20 years old / Yes, the plan is older than 20 years / No, my municipality does not have a coastal zone plan / Don’t know (Check all that apply) | Har kommunen din en kystsoneplan? | Planen er under utvikling / Ja, planen er mellom 0-5 år / Ja, planen er mellom 6-10 år / Ja, planen er mellom 11-20 år / Ja, planen er eldre enn 20 år / Nei, kommunen min har ikke en kystsoneplan / Vet ikke (Velg alle aktuelle) |
| **Status intermunicipal coastal plan** | Does your municipality have an intermunicipal coastal zone plan? | The plan is under development / Yes, the plan is between 0-5 years old / Yes, the plan is between 6-10 years old / Yes, the plan is between 11-20 years old / Yes, the plan is older than 20 years / No, my municipality does not have an intermunicipal coastal zone plan / Don’t know (Check all that apply) | Er kommunen din med i et interkommunalt kystsoneplan samarbeid? | Planen er under utvikling / Ja, planen er mellom 0-5 år / Ja, planen er mellom 6-10 år / Ja, planen er mellom 11-20 år / Ja, planen er eldre enn 20 år / Nei, kommunen min har ikke en interkommunal kystsoneplan / Vet ikke (Velg alle aktuelle) |
