# Supplementary material for: Blue justice: A survey for eliciting perceptions of environmental justice among coastal planners’ and small-scale fishers in Northern-Norway
Source: PLoS One. 2021 May 13;16(5):e0251467. doi: 10.1371/journal.pone.0251467 (PMC8118289; doi:10.1371/journal.pone.0251467)
Supplement: S2 Table — (DOCX) [file pone.0251467.s002.docx]

S2 Table. Blue justice survey to fishers. Questions are arranged in the order they appear for the participant.

|  |  |  |  |  |
| --- | --- | --- | --- | --- |
| *Topic* | *Survey Questions (English)* | *Potential Responses (English)* | *Survey Questions (Norwegian)* | *Potential Responses (Norw.)* |
| **Municipality** | *In this survey you will answer questions about fishing and the use of the coastal zone.*  Which municipality do you want your answers in this survey to refer to?  *Choose the municipality you have the most knowledge about or the strongest attachment to. You can only choose one municipality.* | (drop down list of the 81 coastal municipalities in Northern-Norway) | *I denne undersøkelsen skal du svare på spørsmål om fiske og bruk av kystsonen.*  Hvilken kommune ønsker du at svarene dine baserer seg på?  *Velg den kommunen du har mest kunnskap om eller størst tilknytning til. Du kan kun velge én kommune* | (nedtrekksliste over de 81 kystkommunene i Nord-Norge) |
| **Recognitional justice:**  Knowledge  Culture  Rights | *Focus on your chosen municipality the last 5 years:*  *Regarding decisions about the use of the coastal zone – to what degree has:*   - Fishers’ knowledge been utilized - The significance of the coastal fishing culture been recognized - Matters of importance to the Sea Sami been considered   *To what degree have/has:*   - Younger fishers (18-25 years) had the opportunity to choose fishing as an occupation   *(the latter question is placed among the procedural justice questions in the survey)* | Very small (or no) degree (= --) small degree (= -) / some degree (=N)/ large degree (= +) / very large degree (= ++) / not relevant or don’t know (1-6) | *Fokuser på din valgte kommune de siste 5 årene:*  *Angående beslutninger om bruk av kystsonen - i hvor stor eller liten grad har:*   - Fiskeres kunnskap blitt benyttet - Betydningen av kystfiskekulturen blitt anerkjent - Det blitt tatt hensyn til sjøsamiske forhold   *I hvor stor eller liten grad har:*   - Yngre fiskere (18-25 år) hatt muligheten til å velge fiske som karrierevei   *(det siste spørsmålet er plassert blant prosess spørsmålene i undersøkelsen )* | Svært liten (eller ingen) grad (= --) liten grad (= -) / noen grad (=N)/ stor grad (= +) / veldig stor grad (= ++) / ikke aktuelt eller vet ikke (1-6) |
| **Distributional justice:**  Fish abundance  Access to fish grounds  Habitat  Livelihood  Quality of catch  Fishing effort  Income  Fairness | *Focus on your chosen municipality the last 5 years:*  *To what degree have changes in the use of the coastal zone:*   - Reduced the number of fish and shellfish - Restricted fishers’ access to fishing grounds - Negatively influenced important habitat for fisheries - Reduced the number of fishers - Reduced the quality of fish or shellfish - Increased time, effort and/or travel distance during fishing - Reduced fishers’ income   *To what degree has:*   - The distribution of positive and negative impacts from coastal zone management been fair   *(the latter question is placed among the procedural justice questions in the survey)* | Very small (or no) degree (= --) small degree (= -) / some degree (=N)/ large degree (= +) / very large degree (= ++) / not relevant or don’t know (1-6) | *Fokuser på din kommune de siste 5 årene:*  *I hvor stor eller liten grad har endringer i bruk av kystsonen:*   - Redusert mengde fisk og skalldyr - Begrenset fiskeres tilgang til fiskeområder - Hatt en negativ påvirkningen på viktige habitat for fiskeri - Ført til færre fiskere - Redusert kvaliteten på fisk eller skalldyr - Ført til økt tidsbruk, innsats og/eller reiseavstand under fiske - Redusert fiskeres inntekt   *I hvor stor eller liten grad har:*   - Fordelingen av positive og negative effekter av kystsoneforvaltning vært rettferdig   *(det siste spørsmålet er plassert blant prosess spørsmålene i undersøkelsen)* | Svært liten grad (= --) liten grad (= -) / noen grad (=N)/ stor grad (= +) / veldig stor grad (= ++) / ikke aktuelt eller vet ikke (1-6) |
| **Procedural justice:**  Transparency  Accountability  Influence  Access to justice  Trust  Fairness | *Focus on your chosen municipality the last 5 years:*  *To what degree have/has:*   - Fishers had the opportunity to participate in decisions about the use of the coastal zone - Fishers known who to contact when the use of the coastal zone has caused problems for fisheries - Fishers had influence over decisions about the use of the coastal zone - Conflicts between fishers and other users of the coastal zone been resolved - There been trust between fishers and those in charge of coastal zone planning - The way decisions about the coastal zone has been made been fair | Very small (or no) degree (= --) small degree (= -) / some degree (=N)/ large degree (= +) / very large degree (= ++) / not relevant or don’t know (1-6) | *Fokuser på din valgte kommune de siste 5 årene:*  *I hvor stor eller liten grad har:*   - Det blitt lagt til rette for fiskernes medvirkning i beslutninger om bruk av kystsonen - Fiskere visst hvem de skal kontakte når bruk av kystsonen har ført til problemer for fiskeri - Fiskere hatt innflytelse i beslutninger om bruk av kystsonen - Konflikter mellom fiskeri og andre brukere av kystsonen blitt løst - Det vært tillitt mellom fiskere og de med ansvar for kystsoneplanlegging - Måten beslutninger om bruk av kystsonen har blitt tatt på vært rettferdig | Svært liten grad (= --) liten grad (= -) / noen grad (=N)/ stor grad (= +) / veldig stor grad (= ++) / ikke aktuelt eller vet ikke (1-6) |
| **Challenges for fisheries today** | What are the three greatest challenges for the fisheries in your chosen municipality today? | (participants are asked to list three challenges) | Hva er de tre største utfordringene for fiskeriaktiviteten i din valgte kommune i dag? | (deltakerne bes om å liste tre utfordringer) |
| **Challenges for fisheries in 2050** | In 30 years - what are the three greatest challenges for the fisheries in your chosen municipality then? | (participants are asked to list three challenges) | Om 30 år - hva er de tre største utfordringene for fiskeriaktivitet i din valgte kommune da? | (deltakerne bes om å liste tre utfordringer) |
| **Changes in the use of the coastal zone** | *Focus on your municipality the last 5 years:*   - What changes in the use of the coastal zone have had a negative impact on the fisheries? - What changes in the use of the coastal zone have had a positive impact on the fisheries? | (open question) | *Fokuser på din kommune de siste 5 årene:*   - Hvilke endringer i bruk av kystsonen har hatt negativ innvirkning på fiskeriaktiviteten? - Hvilke endringer i bruk av kystsonen har hatt positiv innvirkning på fiskeriaktiviteten? | (åpent spørsmål) |
|  | Do you have experience with Sea Sami matters in coastal zone management?  *(follow up question placed below the recognitional justice questions in the survey)* | Yes/No | Har du erfaring med sjøsamiske forhold i kystsoneforvaltninga?  *(oppfølgingsspørsmål plassert etter spørsmålene om anerkjennelse)* | Ja/nei |
|  | *If the respondent answers yes to the former question the following question is also asked:*  What are your experiences with the consideration of Sea Sami matters in coastal zone management? | (open question) | *Hvis respondenten svarer ja på forrige spørsmål får vedkomne også spørsmål om:*  Hvilke erfaringer har du med om det tas hensyn til sjøsamiske forhold i kystsoneforvaltninga? | (åpent spørsmål) |
| **Gender** | What is your gender? | Male/female/other or do not wish to respond | Hva er ditt kjønn? | Mann / kvinne / annet eller ønsker ikke å svare |
| **Education** | What is your highest level of education? | No education or not finished primary school / Primary & or secondary school / High School / Vocational training / Higher education: University and College education, lower level (Bachelor's degree) / Higher education: University and College education, higher level (Master's degree, PhD degree, Post graduate university degree) | Hva er din høyeste utdanning? | Ingen utdannelse eller ikke fullført grunnskole / Grunnskole (barneskole, undomsskole, folkeskole, realskole, framhaldsskole) / Videregående skole, gymnas / Fagskole (yrkesrettet alternativ til høyskole eller universitet) / Universitets- og høgskoleutdanning, lavere nivå (inkl. bachelor, cand.mag.) / Universitets- og høgskoleutdanning, høyere nivå (inkl. master, hovedfag og forskerutdanning) |
| **Ethnicity** | Which of these descriptions apply to you? | I am of Sámi decent / I am of Kven decent / I am of Norwegian decent / I am from an EU-country / I am from a non-EU country outside Norway (check all that apply) | Hvilken av disse beskrivelsene passer til deg? (velg alle aktuelle) | Jeg har samisk opphav / Jeg har kvensk opphav / Jeg har norsk opphav / Jeg er fra et EU-land / Jeg er fra et ikke-EU land utenom Norge |
| **Work experience as fisher** | How many years have you worked as a fisher? | (drop down list of values from 0-100) | Hvor mange år har du jobbet som fisker? | (same as English version) |
| **Role on fishing vessel** | What is your role on the fishing vessel? | Crew / quota owner open group / quota owner closed group / other, specify (Check all that apply) | Hvilken rolle har du på fartøyet? | Mannskap / kvoteeier åpen gruppe / kvoteeier lukket gruppe/ annet, spesifiser (velg alle aktuelle) |
| **Species** | Which marine resources do you harvest primarily? | Cod / saithe / haddock / king crab / capelin / Greenland halibut / mackerel / herring / blue whiting / shrimp / redfish / other, please specify (check all that apply) | Hvilke marine ressurser høster du primært? | Torsk / sei / hyse / kongekrabbe / lodde / blåkveite / makrell / sild / kolmule / reker / uer / annet, spesifiser (Velg alle aktuelle) |
| **Fishing gear** | What type of fishing gear do you primarily use? (check all that apply) | Gillnet / Longline / Jigging / Trawl / Danish- or Scottish seine / Purse seine / Other gear (please specify) (check all that apply) | Hvilket/hvilke redskap benytter du primært? | Garn (bunn-, fløyt- og drivgarn) / Line / Jukse / Trål (bunn-, pelagisk-, semipelagisk trål) / snurrevad (Dansk -/ skotsk snurrevad) / not (snurpenot, landnot) / andre redskaper (f.eks. teiner eller ruser) (velg alle aktuelle) |
| **Fishing vessel** | What size is the fishing vessel that you primarily use? | Less than 11 m / 11-14.99 m / 15-20.99 m / 21 – 27.99 m / from 28 m /other (specify) | Hvilken størrelse er fiskefartøyet du primært benytter? | Inntil 11 m / 11-14.99 m / 15-20.99 m / 21 – 27.99 m / fra 28 m / annet (spesifiser) |
| **Income** | What was your average gross annual income, (before tax and other deductions) the last three years? | Less than 270 000 / 270 000-420 000 / 421 000-540 000 / 541 000-660 000 / 661 000-790 000 / 791 000-920 000 / 921 000-1 050 000 / 1 050 000-1 225 000 /  1 225 000-1 500 000 / More than 1 500 000 / don’t know | Hva har din brutto årsinntekt (før skatt og andre fradrag) i gjennomsnitt vært de siste tre årene? | Mindre enn 270 000 / 270 000-420 000 / 421 000-540 000 / 541 000-660 000 / 661 000-790 000 / 791 000-920 000 / 921 000-1 050 000 / 1 050 000-1 225 000 /  1 225 000-1 500 000 / Mer enn 1 500 000 / vet ikke |
| **Resource dependency** | How much your average gross annual income (before tax and other deductions) the last three years have originated from fishing? | 0-10 % /10-20 % /20-30 % / 30-40 % / 40-50 % / 60-70 % / 70-80 % / 80-90 % /90-100 % | Hvor stor andel av din gjennomsnittlige brutto årsinntekt de siste tre årene har du fått fra egen fiskeriaktivitet | 0-10% /10-20 % /20-30 % / 30-40 % / 40-50 % / 60-70 % / 70-80 % / 80-90 % /90-100 % |
| **Resource dependency** | How much of your average gross annual income (before tax and other deductions) the last three years have originated from fishing in the open group fisheries? | 0-10% /10-20 % /20-30 % / 30-40 % / 40-50 % / 60-70 % / 70-80 % / 80-90 % /90-100 % | Hvor stor andel av din gjennomsnittlige brutto årsinntekt de siste tre årene har du fått å fiske i åpen gruppe | 0-10 % /10-20 % /20-30 % / 30-40 % / 40-50 % / 60-70 % / 70-80 % / 80-90 % /90-100 % |
| **Resource dependency** | How much of your average gross annual income (before tax and other deductions) the last three years have originated from fishing in the area close to where you live? | 0-10 % /10-20 % /20-30 % / 30-40 % / 40-50 % / 60-70 % / 70-80 % / 80-90 % /90-100 % | Hvor stor andel av din gjennomsnittlige brutto årsinntekt de siste tre årene har du fått ved å fiske i området der du bor | 0-10 % /10-20 % /20-30 % / 30-40 % / 40-50 % / 60-70 % / 70-80 % / 80-90 % /90-100 % |
| **Resource dependency** | How much of your average gross annual income (before tax and other deductions) the last three years have originated from participating in organized tourist fishing? | 0-10 % /10-20 % /20-30 % / 30-40 % / 40-50 % / 60-70 % / 70-80 % / 80-90 % /90-100 % | Hvor stor andel av din gjennomsnittlige brutto årsinntekt de siste tre årene har du fått ved å delta i organisert turistfiske | 0-10 % /10-20 % /20-30 % / 30-40 % / 40-50 % / 60-70 % / 70-80 % / 80-90 % /90-100 % |
| **Resource dependency** | *To what extent do you agree or disagree with the following:*  I could easily stop fishing and make my living through other means. | Fully disagree (= --) / somewhat disagree (= -) / neutral(=N)/ somewhat agree (= +) / fully agree (= ++) / don’t know (1-6) | *I hvor stor grad er du enig i denne uttalelsen*:  Jeg kan enkelt slutte å fiske og livnære meg på andre måter. | Helt uenig (= --) / uenig (= -) / verken enig eller uenig (=N) / enig (= +) / helt enig (= ++) / Ikke aktuelt for min kommune eller vet ikke (1-6) |
| **Family involvement** | Are other members of your family involved in commercial fishing together with you? | No / Yes, my spouse or partner / Yes, my child(ren) under 18 years / Other, please specify (check all that apply) | Er andre i familien din involvert i kommersiell fiskeriaktivitet sammen med deg? | Nei / Ja, min ektefelle/samboer Ja, egne barn under 18 år / Annet, spesifiser (Velg alle aktuelle) |
